# High-resolution imaging mass spectrometry combined with transcriptomic analysis identified a link between fatty acid composition of phosphatidylinositols and the immune checkpoint pathway at the primary tumour site of breast cancer

**DOI:** 10.1038/s41416-019-0662-8

**Published:** 2019-12-10

**Authors:** Masahiro Kawashima, Mariko Tokiwa, Tomomi Nishimura, Yukiko Kawata, Masahiro Sugimoto, Tatsuki R. Kataoka, Takaki Sakurai, Keiko Iwaisako, Eiji Suzuki, Masatoshi Hagiwara, Adrian L. Harris, Masakazu Toi

**Affiliations:** 1 Department of Breast Surgery, Graduate School of Medicine, Kyoto University, 54 Shogoin-Kawahara-cho, Sakyo-ku, Kyoto 606 8507 Japan; 2 Molecular Oncology Laboratories, Wheaterall Institute of Molecular Medicine, John Radcliffe Hospital, University of Oxford, Oxford, OX3 9DS UK; 3 Health Promotion and Preemptive Medicine, Research and Development Center for Minimally Invasive Therapies, Tokyo Medical University, Sinjuku-ku, Tokyo 160-8402 Japan; 4 Department of Diagnostic Pathology, Graduate School of Medicine, Kyoto University, 54 Shogoin-Kawahara-cho, Sakyo-ku, Kyoto 606-8507 Japan; 5 Department of Target Therapy Oncology, Graduate School of Medicine, Kyoto University, 54 Shogoin-Kawahara-cho, Sakyo-ku, Kyoto 606-8507 Japan; 6 Department of Anatomy and Developmental Biology, Graduate School of Medicine, Kyoto University, Yoshida-Konoe-Cho, Sakyo-ku, Kyoto 606-8501 Japan; 7 Medical Research Support Center, Graduate School of Medicine, Kyoto University, Yoshida-Konoe-Cho, Sakyo-ku, Kyoto 606-8501 Japan

**Keywords:** Breast cancer, Cancer imaging, Cancer metabolism

## Abstract

**Background:**

The fatty acid (FA) composition of phosphatidylinositols (PIs) is tightly regulated in mammalian tissue since its disruption impairs normal cellular functions. We previously found its significant alteration in breast cancer by using matrix-assisted laser desorption and ionisation imaging mass spectrometry (MALDI-IMS).

**Methods:**

We visualised the histological distribution of PIs containing different FAs in 65 primary breast cancer tissues using MALDI-IMS and investigated its association with clinicopathological features and gene expression profiles.

**Results:**

Normal ductal cells (*n* = 7) predominantly accumulated a PI containing polyunsaturated FA (PI-PUFA), PI(18:0/20:4). PI(18:0/20:4) was replaced by PIs containing monounsaturated FA (PIs-MUFA) in all non-invasive cancer cells (*n* = 12). While 54% of invasive cancer cells (*n* = 27) also accumulated PIs-MUFA, 46% of invasive cancer cells (*n* = 23) accumulated the PIs-PUFA, PI(18:0/20:3) and PI(18:0/20:4). The accumulation of PI(18:0/20:3) was associated with higher incidence of lymph node metastasis and activation of the PD-1-related immune checkpoint pathway. Fatty acid-binding protein 7 was identified as a putative molecule controlling PI composition.

**Conclusions:**

MALDI-IMS identified PI composition associated with invasion and nodal metastasis of breast cancer. The accumulation of PI(18:0/20:3) could affect the PD-1-related immune checkpoint pathway, although its precise mechanism should be further validated.

## Background

Matrix-assisted laser desorption and ionisation imaging mass spectrometry (MALDI-IMS) is a novel application that can be used for the precise mapping of lipid distribution in tissue sections.^1^ In MALDI-IMS, mass spectrometry is performed in situ by irradiating ionising laser directly to tissue sections. The histological distribution of ionised molecules is visualised by reconstituting the mass spectra collected with their positional information. The spatial resolution of MALDI-IMS has recently reached <10 μm, which enables the discrimination of small cell clusters within complex histological structures. This makes it possible to analyse small samples, for example, samples acquired through needle biopsy.^2^ In addition, MALDI-IMS does not require the administration of any of the labelling agents to patients prior to sample collection. On the basis of these advantages, MALDI-IMS has been recognised as a promising technology for clinical research and diagnosis.^3,4^

The dynamic molecular weight range in high-resolution analysis of MALDI-IMS is currently from 100 to 1000 Da, and it is suitable for the measurement of phospholipids. Phospholipids are major building blocks of the cellular plasma membrane, and they contain two fatty acids (FAs) in their structure. Differences in FA composition can generate the structural and functional diversity of phospholipids.^5^

Among the major classes of phospholipids, phosphatidylinositols (PIs) have unique characteristics in their functions and FA composition. In mammalian tissue, PIs containing FA(C18:0) and FA(C20:4), abbreviated as PI(18:0/20:4), represent almost half of all PIs, the proportion of which is strictly regulated through unknown mechanisms.^6,7^ Other phospholipid classes exhibit greater diversity in their FA composition, suggesting that the FA composition of PIs could be directly linked to specific biological processes. Recent evidence indicates that the tight regulation of the FA composition of PIs is important for the maintenance of cellular functions, especially the maintenance of major signalling pathways, such as the phosphatidylinositol-3 kinase (PI3K) pathway.^8^ Studies using a prostate cancer model showed that incorporation of docosahexaenoic acid into PI alters PI phosphate and AKT localisation, which in turn affects downstream signalling.^9^ The artificial disruption of the FA remodelling of PIs alters the activity of the PI3K pathway, resulting in an impairment of the asymmetric division of stem cells in *Caenorhabditis elegans* and normal brain development in mice.^10–12^ In addition, in a pancreas cancer model, an oncogenic mutation in *p53* was shown to affect the PI3K pathway via an alteration in PI composition.^13^ As the PI3K pathway is activated frequently in breast cancer,^14^ we previously analysed ten human breast cancer tissues using high-resolution MALDI-IMS and observed the disruption of the tight regulation of the FA composition of PIs in breast cancer cells.^15^ Moreover, we found that the PI composition in breast cancer cells exhibited two distinct features—one showing the significant accumulation of PIs containing monounsaturated FA (PIs-MUFA), namely, PI(16:0/18:1) and PI(18:0/18:1), and the other showing the significant accumulation of PIs containing polyunsaturated FA (PIs-PUFA), namely, PI(18:0/20:3) and PI(18:0/20:4). This preliminary observation suggested that the FA composition of PIs could reflect a certain biological process occurring in breast cancer tissue. Subsequently, other groups identified alterations in PI composition in prostate cancer^16^ and colon cancer models^17^ using high-resolution MALDI-IMS, although the alteration patterns were not completely identical to those observed in breast cancer.

In this study, we analysed the histological distribution of PIs containing different FAs in 56 primary breast cancer tissues. Compatible with our previous observation, breast cancer cells exhibited two distinct features in terms of the balance between PIs-PUFA and PIs-MUFA. The accumulation of PIs-PUFA was observed only in invasive cancer cells and was associated with progressive clinical characteristics. Moreover, combined gene expression array analysis revealed its association with the global activation of tumour immune responses, in particular, the immune checkpoint signalling pathway, suggesting that the dynamism of the FA remodelling of PIs in cancer cells could be a critical molecular process modulating tumour immune reactions.

## Methods

### Sample collection

Breast cancer tissue from primary tumour sites was collected through needle or excisional biopsy at the Department of Breast Surgery, Kyoto University Hospital. Written informed consent was obtained from all patients prior to sample collection. The study protocol was approved by the Ethics Committee for Clinical Research, Kyoto University Hospital (authorisation number G424). The obtained tissue was frozen quickly in liquid nitrogen and stored at −80 °C or in liquid nitrogen until analysis (the storage method of each sample is described in Supplementary Table S1).

### MALDI-IMS

Sample preparation for MALDI-IMS was performed as described previously with minor modifications.^15^ Serial 10-µm sections were mounted on indium–tin-oxide-coated glass slides (Sigma-Aldrich, MO), and were coated with a matrix of 9-aminoacridine hemihydrates (Acros Organics, Geel, Belgium) evaporated at 220 °C for 8 min at a distance of 4 cm from the slides in a vacuum deposition equipment (SVC-700TM/700-2; SANYU ELECTRON, Tokyo, Japan). MALDI-IMS was performed on a high-resolution microscopic imaging mass spectrometer (iMScope; Shimadzu Corp., Kyoto, Japan) under atmospheric pressure.^2^ Normal ductal cells, cancer cells and their surrounding stroma were located in the bright-view mode of this instrument in reference to mapping with serial haematoxylin and eosin (H&E) staining. The pixels for laser ionisation were set at an interval of 7.7 μm. Prior to measurement, the laser was used to irradiate the inter-pixel space ten times with an intensity of 22.5 to minimise the ionisation of non-specific molecules contained in the superficial layer of the matrix. For data acquisition, each targeted pixel was irradiated 80 times with the laser at 800 Hz and an intensity of 18.0. The mass spectra of negatively charged ionised molecules with an *m/z* ranging from 700.0 to 950.0 were collected using an external calibration method. Ion-density maps were created from the acquired mass spectra using Imaging MS Solution software ver.1 (Shimadzu Corp., Kyoto, Japan). Normalisation to the total ion current was conducted to eliminate variations caused by ionisation efficiency.

### Histological analysis

Histological diagnoses were performed with formalin-fixed paraffin-embedded tissue at the Department of Diagnostic Pathology, Kyoto University Hospital. Histological grade was determined according to the modified Bloom–Richardson system. Positivity for ER, PgR and Her2 was determined according to the American Society of Clinical Oncology/College of American Pathologist Guidelines.^18^ The 9-aminoacridine hemihydrate matrix was removed from the slides by dipping them in methanol for 30 s. Subsequently, the slides were stained with H&E. Using this H&E staining, the senior pathologist determined the areas occupied by normal ductal cells, non-invasive cancer cells, invasive cancer cells and stroma in mass imaging. To maximise assay sensitivity, we focused on ten pre-screened PIs that were detectable within breast sections in our previous study. As described previously, the signals appearing at *m/z* 807.5, 809.5, 833.5, 835.5, 837.5, 861.5, 863.5, 885.5, 887.5 and 889.5 were recognised as the signals from PI(16:0/16:1), PI(16:0/16:0), PI(16:0/18:2), PI(16:0/18:1), PI(16:0/18:0), PI(18:0/18:2), PI(18:0/18:1), PI(18:0/20:4), PI(18:0/20:3) and PI(18:0/20:2), respectively.^15^ The mass intensities were normalised to the sum of the intensity of the ten PIs to perform intra- and inter-sectional comparisons. Tumour-infiltrating immune cells were assessed according to the International Working Group Recommendations.^19^

### Statistical analysis

For the analysis of numerical data, a Mann–Whitney test or two-way analysis of variance (ANOVA) with Sidak’s post hoc multiple comparisons test were carried out on Prism 6 (GraphPad Software, Inc., CA). Chi-squared tests were performed to test the association between the FA composition of PIs and the clinicopathological parameters in univariate analysis. Subsequently, a multiple logistic regression model was applied to identify the independently associated variables. “Age,” “menopausal status” and “body mass index” were included as variables that could affect tumour lipid metabolism. Univariate and multivariate analyses were performed using JMP ver.10 (SAS Institute, Inc., NC). Differences were considered statistically significant at *p* < 0.05. The original data for these analyses are provided in Supplementary Table S1.

### RNA extraction and gene expression array

The residual samples that were used for the MALDI-IMS measurements were put into an RNA extraction buffer (Buffer RLT of an RNeasy Mini Kit, Cat#74140; QIAGEN, Tokyo, Japan) containing zirconia (φ 1.0 mm) and stainless-steel beads (φ 5.5 mm). The samples were homogenised by vigorous shaking three times at 3000 rpm for 15 s at 4 °C and subsequently by shaking two times at 3500 rpm for 15 s at 4 °C with Bead Smash12 (WKN-BS-12R; WAKENBTECH, Kyoto, Japan). The quality of extracted RNA was evaluated using an RNA integrity number measured with an Agilent RNA6000 Nano Kit (Agilent Technologies Japan, Tokyo, Japan). Gene expression analysis was performed with a GeneChip® Human Genome U133 Plus 2.0 Array (Cat# 900470; Affymetrix, CA) at the Medical Research Support Center, Graduate School of Medicine, Kyoto University, which was supported by Platform for Drug Discovery, Informatics and Structural Life Science from the Ministry of Education, Culture, Sports, Science and Technology, Japan.

### Pathway enrichment analysis of breast tumours

The gene expression data were normalised through the MAS5 algorism.^20^ Pearson’s correlation coefficient was calculated between the expression value of individual genes and the ratio of PIs-PUFA to PIs-MUFA in cancer cells, and the genes whose absolute correlation coefficient values >0.41 (*α* < 0.01 in two-tailed probability) were selected (a full report on the correlation coefficient and gene expression values is shown in Supplementary Table S2). The mean log2-transformed expression of these genes was compared between the tumours with and without histological PI(18:0/20:3) accumulation. Pathway enrichment analysis was performed with Metacore^TM^ (Thomson Reuters, NY) by using the genes whose mean expression levels showed a significant difference (Student’s *t* test, two-tailed, *p*-value < 0.05) between the tumours with and without histological PI(18:0/20:3) accumulation. The pathways with a *p*-value < 0.001 were selected as significant pathways.

### Hierarchal clustering and co-occurrence/mutual exclusivity tests

The genes categorised in the pathway map of “breast cancer” and “inhibitory PD-1 signalling in T cells” and the potential genes related to FA and PI metabolism were selected from the gene set used in the pathway enrichment analysis. Hierarchical clustering using Pearson’s correlation coefficient was performed with MultiExperiment Viewer software (version 4.9.0; Dana-Farber Cancer Institute, MA) by using log2-transformed expression data. Co-occurrence and mutual exclusivity tests were performed on cBioPortal (version 1.3.2; Memorial Sloan Kettering Cancer Center, NY) using the METABRIC and TCGA breast cancer data sets.^14,21–24^

### Cell culture and gene silencing by RNA interference

A breast cancer cell line, HCC1806, was purchased from the American Type Culture Collection. Cells with and without FABP7 knockdown were established by the lentiviral transduction system using particles containing a FABP7 short-hairpin RNA (shRNA) expression cassette (Mission® shRNA, Sigma-Aldrich, TRCN0000059744) or a non-targeting shRNA sequence (SHC002U), respectively. Cells expressing the shRNA were selected in puromycin (Invitrogen)-containing medium (2 μg/mL). The cells were maintained in Dulbecco’s modified Eagle’s medium (10 mM glucose) (Gibco) supplemented with 10% fetal bovine serum (FBS) in a humidified incubator with 5% CO_2_ at 37 °C.

### RNA sequencing and pathway enrichment analysis of HCC1806

RNA was isolated using the TRIzol® Reagent (Invitrogen), and its integrity was assessed using a Bioanalyzer (Agilent). RNA sequencing was performed as previously described^25^ (complete gene expression data is shown in Supplementary Table S3). Pathway enrichment analysis was performed with MetacoreTM (Thomson Reuters, NY).

### Liquid chromatography–mass spectrometry (LC–MS)

Lipids were extracted from cell pellets through Bligh and Dyer method. Nexera UHPLC system equipped with LCMS-8050 (Shimadzu Corp., Kyoto, Japan) was used for chromatographic separation and detection of PIs. Separation was performed at 45 °C using an analytical column, Kinetex C8 (2.1 mm id × 150 mm length, 2.6 µm) (Phenomenex). The gradient mobile phase system consisted of 20 mM ammonium formate (mobile phase A) and 50% acetonitrile, 50% 2-propanol (mobile phase B). ESI-negative mode was used for the detection of PIs. PI(12:0/13:0) (Merck) was added as an internal standard for calculating PI concentrations.

### Flow cytometry

Cells were suspended in 100 μl of PBS containing 1% FBS and labelled with 2 μl of APC anti-human CD274 (B7–H1, PD-L1) antibody (clone 29E.2A3, Biolegend) for 30 min on ice. Cells were then washed twice with PBS containing 1% FBS, and fluorescence was analysed using an Attune NxT Flow Cytometer (Thermo Fisher).

## Results

### Availability of samples and validity of MALDI-IMS for clinically acquired breast tissue

**Fig. 1:**
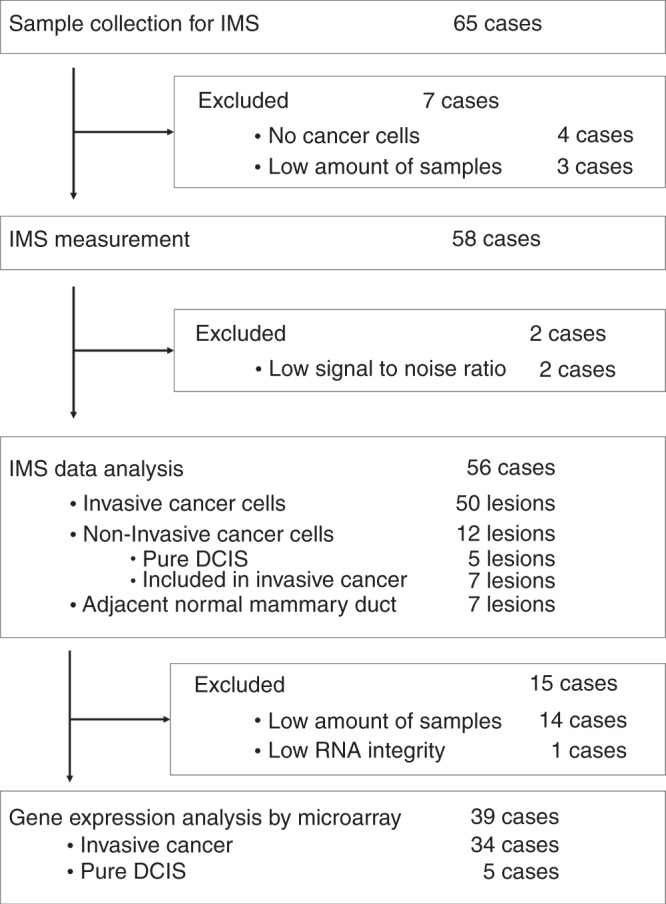
Overview of the study procedure.

Primary breast cancer tissue was collected from 65 patients with breast cancer (Fig. 1). During sample preparation, seven cases were excluded because of insufficient sample volume (*n* = 3) and the lack of cancer cells within the sections (*n* = 4). Samples acquired from 58 patients (89%) were applicable for MALDI-IMS measurement. Two patients were excluded from analysis after MALDI-IMS measurement because of poor imaging quality, which might have been caused by the degradation of target molecules during storage. As a result, the tissues obtained from 56 patients (86% of all patients; 96% of the measured patients), which were composed of 50 lesions of invasive cancer cells, 12 lesions of non-invasive cancer cells and 7 lesions of normal ductal cells, were subjected to histological analysis. With regard to the non-invasive cancer cells, five lesions were from pure ductal carcinoma in situ (DCIS) without an invasive component and seven lesions were from invasive carcinoma. Gene expression arrays could be applied to 39 residual samples (70% of all analysed cases) with a sufficient volume for subsequent RNA extraction. The quantitative and imaging performance of MALDI-IMS was tested using breast cancer tissue obtained from patient-derived xenografts. MALDI-IMS clearly illustrated the difference in PI distribution between human breast cancer cells and the surrounding stroma (Supplementary Fig. S1a). In addition, its quantitative performance was confirmed to be equal to that of LC–MS analysis (Supplementary Fig. S1b).

### Accumulation of PIs-MUFA is a common feature of non-invasive breast cancer cells

**Fig. 2:**
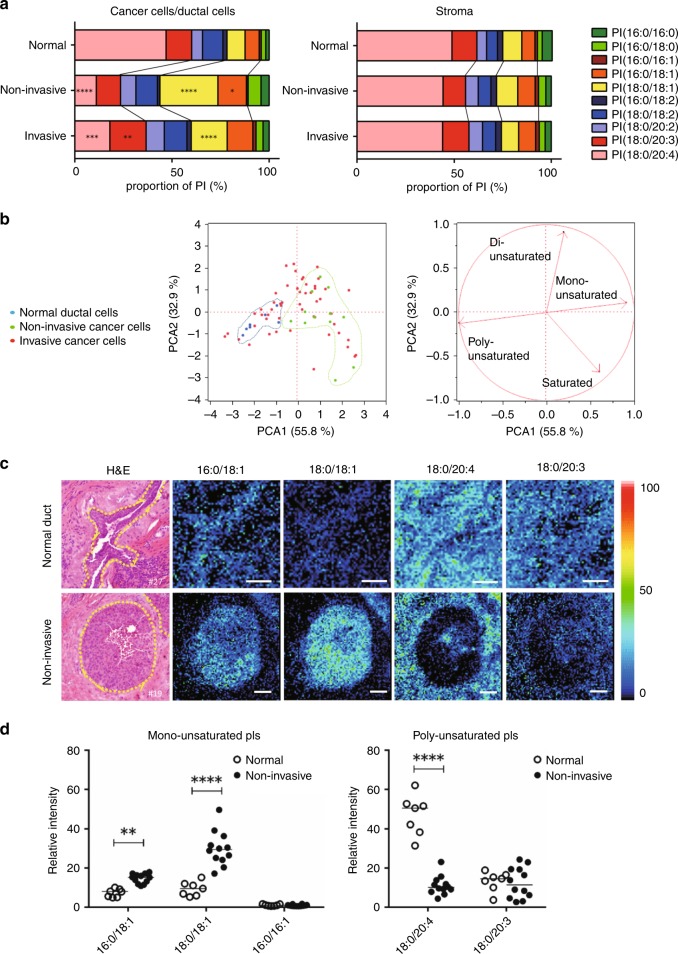
Replacement of PI(18:0/20:4) with PIs-MUFA as a common process of in situ breast cancer growth. **a** Left: the averaged FA composition of PIs detected in cancer cells and adjacent normal ductal cells. Right: the averaged FA composition of PIs detected in the surrounding stromal areas. * indicates a statistically significant difference in the comparison between normal ductal cells and non-invasive cancer cells or between non-invasive cancer cells and invasive cancer cells. Saturated, monounsaturated, di-unsaturated and polyunsaturated PIs are coloured in blue, yellow, red and green, respectively. Two-way ANOVA with Sidak’s post hoc multiple comparisons test. **p < 0.01; ***p < 0.001; ****p < 0.0001. **b** Principal component analysis of normal ductal cells and cancer cells based on the proportion of ten PIs. Left: each dot indicates an individual lesion. Blue: normal ductal cells; green: non-invasive cancer cells; red: invasive cancer cells. Right: the arrows indicate the contribution rate of PIs with different degrees of unsaturation. The contribution ratio of each component is displayed on the axis. **c** Representative histological mapping of PIs and the corresponding H&E staining. Upper panels: images of normal ductal cells and their associated stroma. Lower panels: images of non-invasive cancer cells and stroma. Normal ductal cells and cancer cells are encircled by a yellow dotted line in H&E staining. Relative intensity of ion signals is represented by an RGB scale. Scale bar (white): 50 μm. **d** Comparison of relative intensities between non-invasive cancer cells and normal ductal cells. White and black circles show individual lesions of normal ductal and non-invasive cancer cells, respectively. * indicates a statistically significant difference in the comparison between normal and non-invasive cancer cells. Two-way ANOVA with Sidak’s post hoc multiple comparisons test. **p < 0.01; ****p < 0.0001.

Figure 2a shows PI composition averaged across all analysed lesions. PI(18:0/20:4)—PIs carrying FA(18:0) and FA(C20:4)—represented almost half of all PIs both in normal ductal cells and in their associated stroma (Fig. 2a).

This predominance of PI(18:0/20:4) was similarly observed in all stromal areas surrounding the cancer cells (Fig. 2a, right). The stromal areas included in the analysis consisted mainly of fibroblasts and immune cells, and the proportion of immune cells did not affect the stromal PI composition (Supplementary Fig. S2a). In contrast, a significant alteration in the averaged composition of PIs was observed in cancer cells. In cancer cells, the proportion of PI(18:0/20:4) was significantly decreased, while the proportion of the other PIs was increased (Fig. 2a, left). Importantly, the averaged proportion of two PIs-MUFAs, PI(16:0/18:1) and PI(18:0/18:1), was significantly increased in non-invasive cancer cells. In principal component analysis, non-invasive cancer cells formed an independent cluster that was clearly separated from that of normal ductal cells (Fig. 2b). The cluster of non-invasive cancer cells was located on the opposite side of normal ductal cells, suggesting that non-invasive cancer cells had an increased ratio of PIs-MUFA to PIs-PUFA when compared with normal ductal cells (Fig. 2b).

Compatible with these findings, the PIs-MUFA accumulated densely in non-invasive cancer cells in histological images (Fig. 2c). Although PIs-MUFA could accumulate in normal ductal cells to some extent, their intensity was much lower than that observed in non-invasive cancer cells (Fig. 2c, d). In clear contrast to the distribution of PIs-MUFA, PI(18:0/20:4) became almost undetectable in non-invasive cancer cells (Fig. 2c). This complementary pattern between PIs-MUFA and PI(18:0/20:4) was observed in all non-invasive cancer cells (Fig. 2d). PI(16:0/16:1) and PI(18:0/20:3), components of PIs-MUFA and PIs-PUFA, respectively, did not exhibit significant differences between normal ductal cells and cancer cells (Fig. 2c, d).

### Accumulation of PIs-PUFA is a hallmark of invasive cancer cells

In the score plot of principal component analysis, invasive cancer cells deviated to the more unsaturated side compared with non-invasive cancer cells (Fig. 2b). This suggested that the PIs in invasive cancer cells have a higher degree of unsaturation compared with PIs in non-invasive cancer cells. Pure DCIS lesions tended to have a greater proportion of saturated PIs compared with normal ductal cells, and non-invasive cancer cells existed in invasive diseases (Supplementary Fig. S2b). In a comparison between non-invasive and invasive cancer cells co-existing in the same tumour sections, the proportion of saturated and monounsaturated PIs was decreased in invasive cancer cells compared with matched non-invasive cancer cells (*p* = 0.0196 and 0.0393, respectively) (Supplementary Fig. S2b). On the other hand, the proportion of di-unsaturated and polyunsaturated PIs tended to increase in invasive cancer cells (*p* = 0.0027 and 0.0992, respectively) (Supplementary Fig. S2b). These findings indicated that cancer cells acquired less-unsaturated PIs at the in situ growth stage and then increased the degree of unsaturation in accordance with their invasion.

**Fig. 3:**
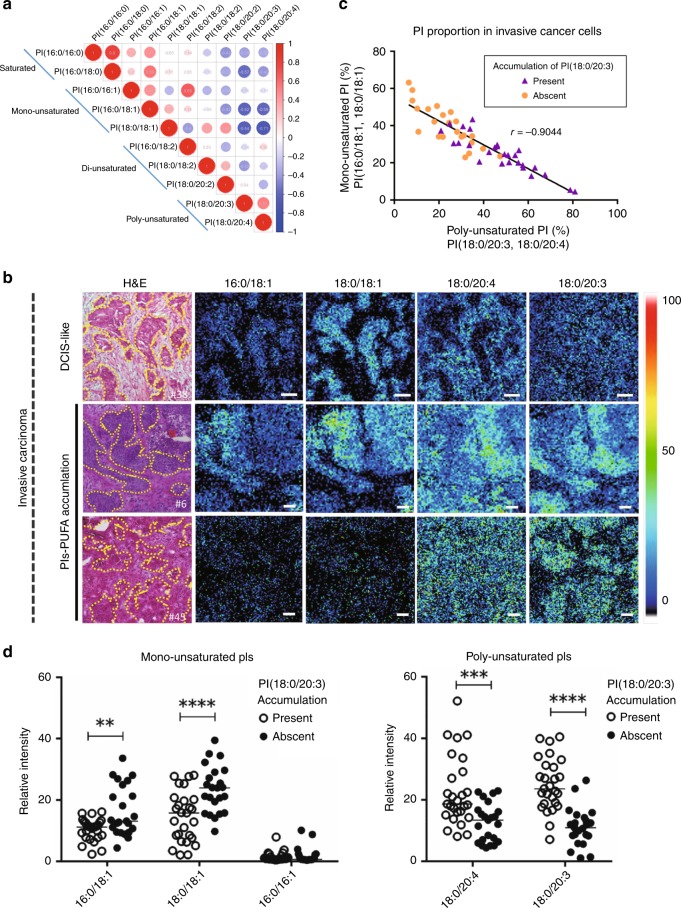
Replacement of PIs-MUFA with PIs-PUFA occurs in invasive cancer cells and is recognised as PI(18:0/20:3) accumulation. **a** Inter-individual correlation of PIs with different FA compositions in invasive cancer cells. Red and blue circles indicate a positive and negative correlation, respectively. The circled values show the correlation coefficient of each combination, which is also represented by circle size. **b** Representative histological mapping and the corresponding H&E staining of invasive cancer cells and their surrounding stroma. Cancer cells are encircled by a yellow dotted line in H&E staining. “N” indicates a necrotic region. Relative intensity of ion signals is represented by an RGB scale. Scale bar (white): 50 μm. **c** Correlation of PIs-MUFA and PIs-PUFA in invasive cancer cells. Triangles and circles indicate individual lesions of invasive cancer cells. The presence of PI(18:0/20:3) accumulation in cancer cells is indicated as a purple triangle. **d** Comparison of the intensities of invasive cancer cells between the presence and absence of PI(18:0/20:3) accumulation. White and black circles indicate individual lesions for the presence or absence of PI(18:0/20:3) accumulation in cancer cells, respectively. * indicates a statistically significant difference in the comparison between normal and DCIS. Two-way ANOVA with Sidak’s post hoc multiple comparisons test. **p < 0.01; ***p < 0.001; ****p < 0.0001.

In a comparison across patients with invasive disease, two PIs-PUFAs, PI(18:0/20:3) and PI(18:0/20:4), showed the strongest inverse correlation with two PIs-MUFAs, PI(16:0/18:1) and PI(18:0/18:1), in invasive cancer cells (Fig. 3a). Compatible with these findings, invasive cancer cells showed two distinct patterns in terms of the balance between PIs-MUFA and PIs-PUFA in histological imaging; ones showing a similar pattern with non-invasive cancer cells in which PIs-MUFA significantly accumulated in cancer cells, and the others showing a significant accumulation of PIs-PUFA instead of PIs-MUFA (Fig. 3b). In the latter cases, PI(18:0/20:3) and PI(18:0/20:4) increased simultaneously in invasive cancer cells. However, the location of invasive cancer cells was more easily recognisable by PI(18:0/20:3) accumulation than PI(18:0/20:4) accumulation, since PI(18:0/20:4) also existed abundantly in the surrounding stroma (Fig. 3b). The cancer cells linearly reduced the proportion of PIs-MUFA in accordance with the increase of PIs-PUFA (Fig. 3c). The accumulation of PI(18:0/20:3) in histological imaging was well correlated with the increased ratio of PIs-PUFA/PIs-MUFA in cancer cells (Fig. 3c, d). Therefore, we decided to use the presence of “PI(18:0/20:3) accumulation” in histological imaging as the objective threshold to define “PIs-PUFA accumulation.”

### Histological accumulation of PI(18:0/20:3) in invasive cancer cells indicates the presence of lymph node metastasis

The histological accumulation of PI(18:0/20:3) in cancer cells was observed in 54% of cases with invasive cancer (27 out of 50; Table 1). Among the tested clinicopathological parameters, “age over 60 years,” “postmenopausal state,” “presence of lymph node metastasis,” “negativity for oestrogen receptor (ER)” and “negativity for progesterone receptor (PgR)” were positively associated with PI(18:0/20:3) accumulation (Table 1). Histological PI(18:0/20:3) accumulation was observed more frequently in ER-positive/PgR-negative/human epidermal growth factor receptor 2 (Her2)-negative and triple-negative subtypes, although the difference did not reach statistical significance (Table 1). The sample storage method was not associated with histological PI(18:0/20:3) accumulation (Table 1). In univariate and multivariate analyses, histological PI(18:0/20:3) accumulation was confirmed as an independent factor that could predict the presence of lymph node metastasis (Table 2). This suggested that histological PI(18:0/20:3) accumulation in invasive cancer cells occurred in more progressive disease with lymph node metastasis.

**Table 1:** Correlation between histological accumulation of PI(18:0/20:3) in invasive cancer cells and clinicopathological parameters.

Variable	Histological PI(18:0/20:3) accumulation		*P*-value
	Present	Absent	
Total number of cases	27	23	
Age*			
<60	7	14	<0.05
60≦	20	9	
Menopause*			
Pre	4	10	<0.05
Post	23	13	
*BMI*			
<20	8	8	0.91
20≦ , < 23	12	9	
23≦	7	6	
*Histological grade*			
1, 2	16	17	0.27
3	11	6	
*pT*			
1, 2	23	18	0.53
3, 4	4	5	
Lymph node metastasis*			
Negative	8	14	<0.05
Positive	18	9	
Unknown	1	0	
*Distant organ metastasis*			
Negative	23	22	0.20
Positive	4	1	
*Stage*			
I, II	6	7	0.51
III, IV	21	16	
ER*			
Negative	11	3	<0.05
Positive	16	20	
PgR*			
Negative	16	4	<0.01
Positive	11	19	
*Her2*			
Negative	24	19	0.52
Positive	3	4	
*Tumour subtypes*			
ER+/PgR+/Her2−	11	16	0.05
ER+/PgR−/Her2−	3	1	
Any ER and PgR/Her2+	3	4	
ER–/PgR−/Her2−	10	2	
*Infiltrating immune cells*			
Under 10%	14	14	0.52
10% or over	13	9	
*Storage method*			
−80 °C	14	12	0.98
Liquid nitrogen	13	11	

* indicates variables which are statistially significant

**Table 2:** Univariate and multivariate logistic regression analysis predicting the presense of lymph node metastasis

Variable	Univariate		Multivariate	
	Odds ratio	95% confidence interval	Odds ratio	95% confidence interval
PI(18:0/20:3) accumulation*	3.50*	1.09–11.21	7.88*	1.64–59.30
PgR negative	0.71	0.22–2.22	0.093*	0.0068–0.81
Age 60≦	1.70	0.55–5.25	3.61	0.53–34.5
Postmenopause	0.89	0.26–3.02	0.23	0.018–1.90
ER negative	1.12	0.33–3.78	4.90	0.57–54.80

* indicates variables that are statistially significant

### Histological accumulation of PI(18:0/20:3) in invasive cancer cells is associated with the activation of the programmed cell death-1 (PD-1)-related immune checkpoint pathway

**Fig. 4:**
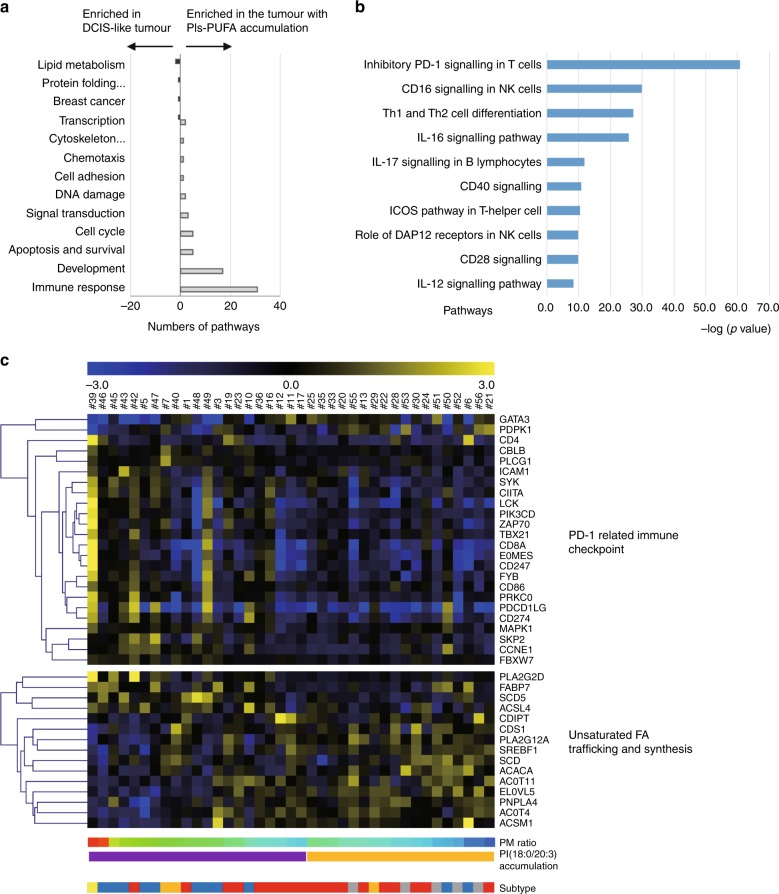
Molecular pathways associated with PI-PUFA accumulation in invasive cancers. **a** Gene ontology of the pathways differentially activated in the tumours with or without PI(18:0/20:3) accumulation. **b** The pathways related to the immune response that are differentially activated in invasive breast tumours with or without PI(18:0/20:3) accumulation. “–Log (*p*-value)” of each pathway is shown. **c** A heatmap illustrating the expression of individual genes related to “inhibitory PD-1 signalling in T cells” (upper) and “unsaturated fatty acid trafficking and synthesis” (lower). Gene expression values are indicated by a blue to yellow colour scale (top bar). The presence or absence of PI(18:0/20:3) accumulation in histological images is shown in purple and yellow, respectively. The values of the PM ratio are indicated by a rainbow colour scale (bottom bar). Tumour subtypes are shown in red (luminal), blue (triple negative), orange (luminal Her2), yellow (Her2) and grey (pure DCIS).

To explore the biological process related to the observed remodelling of PIs in cancer cells, pathway enrichment analysis was performed using 1112 genes that were differentially expressed between the tumours with and without histological PI(18:0/20:3) accumulation. The tumours for this analysis consisted of 19 luminal, 11 triple-negative, 4 Her2-enriched and 5 pure DCIS cases (Fig. 1). The analysis identified 76 pathways that were associated with histological PI(18:0/20:3) accumulation in cancer cells (Supplementary Table S4). The categories of these pathways clearly showed that various types of immune reactions were differentially activated in tumours with PI(18:0/20:3) accumulation (Fig. 4a; Supplementary Table S4). Among these immune reactions, the PD-1-related immune checkpoint pathway showed the highest correlation with histological PI(18:0/20:3) accumulation (Fig. 4b, c). This indicated that cancer cells with a higher amount of PI(18:0/20:3) inhibited host antitumour immunity by activating the immune checkpoint pathway, while multiple immune responses were activated concomitantly in the tumour microenvironment.

### Fatty acid-binding protein 7 regulates PI(18:0/20:3) accumulation and the PD-1-related immune checkpoint pathway

The pathway enrichment analysis identified that two pathways related to de novo fatty acid synthesis were enriched in DCIS-like tumours (Fig. 4a; Supplementary Table S4). This is consistent with our earlier observation that saturated PIs tended to be higher in non-invasive lesions (Fig. 2b; Supplementary Fig. S2b). However, no lipid metabolic pathways were enriched in relation to PUFA accumulation. Thus, the association between the expression profiles of lipid metabolic genes and the histological accumulation of PI(18:0/20:3) in cancer cells was tested by using hierarchical clustering (Supplementary Table S5). Multiple genes involved in FA degradation through both β-oxidation and peroxidation were downregulated in tumours with PI(18:0/20:3) accumulation (Supplementary Fig. S3). In addition, multiple genes involved in glycosylphosphatidylinositol biosynthesis, where the incorporation of MUFAs into PIs could be increased, were downregulated in tumours with PI(18:0/20:3) accumulation (Supplementary Fig. S3). In clear contrast, the genes involved in FA desaturation (stearoyl-CoA desaturase, *SCD5*) and PUFA trafficking (fatty acid-binding protein 7, *FABP7*; acyl-CoA synthetase 4, *ACSL4*; phospholipase A2G2D, *PLA2G2D*) were upregulated in tumours with PI(18:0/20:3) accumulation (Fig. 4c). Among these four genes, the expression of *FABP7*, which is involved in PUFA transport (26), was correlated with the expression of PD-L1/PD-L2 in both the METABRIC and TCGA data sets (Tables 3, 4, respectively).

**Table 3:** Co-occurrence and mutual exclusivity test in METABRIC data set (*n* = 2509).

	Metabolic gene	Log OR	*P*-value	Association
*CD274 (PD-L1)*	FABP7*	1.11	4.47E–05	Co-occurrence
	ACSL4*	1.01	3.56E–03	Co-occurrence
	SCD5*	0.87	9.26E–03	Co-occurrence
	*PLA2G2D*	−0.07	0.557	Mutual exclusivity
*PDCD1LG2 (PD-L2)*	FABP7*	1.31	1.23E–06	Co-occurrence
	ACSL4*	1.54	2.14E–06	Co-occurrence
	SCD5*	0.73	0.0362	Co-occurrence
	*PLA2G2D*	0.00	0.616	Mutual exclusivity

* indicates the correlation of statistically significant derived from Fisher exact test

**Table 4:** Co-occurrence and mutual exclusivity test in TCGA data set (*n* = 825).

	Metabolic gene	Log OR	*P*-value	Association
*CD274 (PD-L1)*	FABP7*	1.91	1.44E–05	Co-occurrence
	PLA2G2D*	2.81	5.82E–08	Co-occurrence
	*SCD5*	0.63	0.244	Co-occurrence
	*ACSL4*	<−3	0.898	Mutual exclusivity
*PDCD1LG2 (PD-L2)*	FABP7*	1.24	1.59E–02	Co-occurrence
	PLA2G2D*	2.39	3.35E–05	Co-occurrence
	*SCD5*	0.36	0.428	Co-occurrence
	*ACSL4*	<−3	0.915	Mutual exclusivity

* indicates the correlation of statistical significance derived from Fisher exact test

**Fig. 5:**
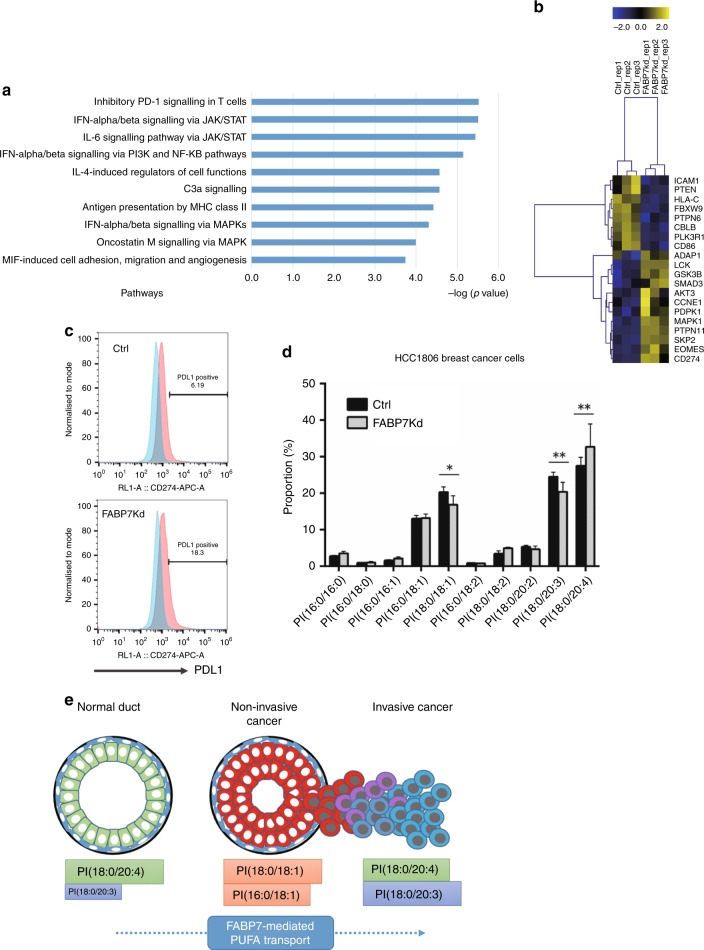
FABP7 knockdown induces the alteration of PD-1-related gene expression profile and PI composition in HCC1806 cells. **a** The pathways related to the immune response that are differentially activated in HCC1806 cells with or without FABP7 knockdown. The “–Log (*p*-value)” of each pathway is shown. **b** A heatmap illustrating the expression of individual genes related to “inhibitory PD-1 signalling in T cells”. Gene expression values are indicated by a blue to yellow colour scale (top bar). The analysis was performed in biological triplicate. **c** Flow cytometry analysis of PD-L1 expression. The x axis represents PD-L1 expression. The PD-L1-positive population was determined relative to the negative control peak (blue). **d** The difference of FA composition of PIs between HCC1806 cells with and without FABP7 knockdown. * indicates a statistically significant difference in the comparison two-way ANOVA with Sidak’s post hoc multiple comparisons test. **p < 0.01; ***p < 0.001; ****p < 0.0001. **e** The graphical abstract of this study.

Therefore, we compared gene expression profile and PI composition between HCC1806 breast cancer cells with specific knockdown of FABP7 and a control (knockdown efficiency is shown in Supplementary Fig. S4). FABP7 knockdown in HCC1806 breast cancer cells significantly altered the expression of genes involved in the PD-1-related immune checkpoint pathway among the pathways related to immune responses (Fig. 5a, b; Supplementary Table S6). Having shown upregulation of PD-L1 (CD274) in our RNA-seq data, we performed flow cytometry and confirmed that PD-L1 expression was increased in FABP7-knockdown cells (Fig. 5c). In addition, FABP7 knockdown significantly altered the proportion of PI(18:0/18:1), PI(18:0/20:3) and PI(18:0/20:4) (Fig. 5d). The proportions of PI(18:0/18:1) and PI(18:0/20:3) decreased, and the one of PI(18:0/20:4) increased; the PI proportion of FABP7-knockdown cells becomes similar with the one of non-malignant cells. Notably, uptake of both MUFAs and PUFAs was equal between FABP7-knockdown and control cancer cells (Supplementary Fig. S5a). In addition, of the genes related to unsaturated fatty acid synthesis and transport, we found that ACSL4 was upregulated upon FABP7 knockdown (Supplementary Fig. S5b). Since ACSL4 is involved in PUFA transport,^26^ its upregulation could be a compensatory process to the knockdown of FABP7 function. These results clearly show that FABP7 regulates PD-L1 expression and PI composition in cancer cells. However, it also implies that the coordination of multiple genes related to PUFA synthesis and transport could be essential in determining PI composition and the direction of the modulation of the immune checkpoint pathway. In summary, the findings from clinical samples suggested that FABP7 was a key molecule that contributed to the accumulation of PI(18:0/20:3), as well as the regulation of the immune checkpoint pathway in primary breast cancer. Its underlying molecular mechanism will be further elucidated (Fig. 5e).

## Discussion

The model for the sequential conversion of lipid metabolites was proposed a decade ago; however, it has not been confirmed using clinically acquired cancer tissues.^27^ In this study, we described the dynamic change of the FA composition of PIs in breast cancer cells in accordance with disease progression. Normal ductal cells and their stroma always contain a single PI-PUFA, PI(18:0/20:4), as the major component. While this strict homoeostasis of the FA composition of PIs was maintained in cancer-associated stroma, all non-invasive cancer cells lost the predominance of PI(18:0/20:4) and acquired two PIs-MUFAs, PI(16:0/18:1) and PI(18:0/18:1) (Fig. 5c). This suggested that the accumulation of PIs-MUFA was a common metabolic process that occurred during in situ growth. In addition, PIs containing saturated FAs tend to be higher in pure DCIS lesions, although this was not statistically significant (Fig. 2b; Supplementary Fig. S2). The increase in saturated and monounsaturated PIs in non-invasive lesions is likely due to de novo fatty acid synthesis, since pathway enrichment analysis showed that the pathway is enriched in invasive cancers that exhibit a DCIS-like PI composition (Fig. 4a). Fatty acid synthesis is known to be generally upregulated in malignant cells.^27–30^ The accumulation of PIs-MUFA was maximised in non-invasive cancer cells and retained in about half of invasive cancer cells. However, they were replaced by two PIs-PUFAs, PI(18:0/20:3) and PI(18:0/20:4), in the other invasive cancer cells (Fig. 5c). The accumulation of PIs-PUFA was observed most frequently in cancer cells with lymph node metastasis, suggesting that the acquisition of PIs-PUFA by cancer cells could be essential for stromal invasion and subsequent lymph node metastasis. The accumulation of PI(18:0/20:3) is likely to be a key feature of invasive cancer cells since normal ductal cells solely accumulated PI(18:0/20:4) instead of PI(18:0/20:3).

The degree of unsaturation of phospholipids has been reported to be associated with an immune reaction during ischaemia,^31^ traumatic cellular injury,^32^ cancer cell homing^33^ and neural cell maturation.^34^ Intriguingly, our combined gene expression analysis revealed the strong correlation between the FA composition of PIs and immune-related pathway. In addition, the analysis also revealed that PUFA trafficking conducted by multiple genes like *FABP7* could contribute to PI remodelling in breast cancer. FABPs are cytosolic proteins that can uptake external FAs and transport them to various subcellular compartments.^35^ FABP7 has a higher affinity for PUFAs than other FABP family proteins, and it sustains brain development by supplying PUFAs that are essential for this process.^36^ Since FABP7 knockdown in the breast cancer cells leads to the alteration of PI composition and the gene expressions of PD-1-related immune checkpoint pathway, targeting PUFA trafficking mediated by FABP7 is likely to enhance the effect of immune checkpoint inhibition. However, it remains unclear whether FABP7 positively or negatively regulates the immune checkpoint pathway in the tumour microenvironment, since PD-L1 was upregulated in cancer cells upon FABP7 knockdown. It should be noted that ACSL4 is also upregulated in FABP7-knockdown cells, suggesting that ACSL4 could compensate for FABP7 function. ACSL4, which preferentially transports polyunsaturated fatty acyl CoA, was reported to regulate PI composition^26^ and may be associated with breast cancer invasiveness.^37^ The compensation could explain why the absolute differences of PI composition are relatively small. In our in vitro experimental model, we employed a stable knockdown system using shRNA to minimise damage to the lipid membrane. The ability of cells to adapt during the selection period could minimise observed differences in PI composition.

This study showed that ACSL4, PLA2G2D and SCD5 could also be responsible for the FA remodelling of PIs. PLA2G2D, a secretory-type phospholipase preferentially expressed in lymphoid tissue, regulates PUFA concentration in the tumour microenvironment and modulates immune reaction.^38^ We performed quantitative PCR using multiple breast cancer cell lines and found that PLA2G2D was not expressed in any of them (data not shown). It is known that PLA2G2D is expressed mainly in lymphoid tissue. Thus, it should also be expressed in stromal cells and causes the release of PUFAs into tumour-associated vasculature. SCD5 generates MUFA by desaturating FAs by adding a double bond at the delta-9 position of saturated FAs, and its inhibition was shown to have an antitumour effect.^39,40^ To synthesise PUFA from MUFA, subsequent desaturation by delta-3 or delta-6 desaturase (abbreviated as FADS1/2) is required. Since FADS1/2 was not found to be differentially expressed in our study, we could posit that PUFAs transferred from an intracellular lipid source (i.e. lipid droplets or other phospholipid species), or extracellular circulation by FABP7 or ACSL4 could be incorporated into PIs. Therefore, it would be interesting to elucidate how the coordination of FABP7, ACSL4 and PLA2G2D in tumour microenvironment modulates the remodelling of membrane lipids as well as tumour immunity.

It has been reported that membrane lipid composition could affect the interaction between cancer cells and immune cells. Several glycolipids, including PIs, function as the essential backbone of antigen presentation to activate natural killer T cells. Glycolipid metabolites also function as “lipid antigens”.^41–43^ Importantly, the alteration of the glycolipid MUFA/PUFA ratio in a leukaemic T-cell line was shown to modulate release from the inositol 1,4,5-trisphosphate-sensitive Ca^2+^ store.^44^ This evidence also supports the findings of this study. Since the advantage of blocking PD-1 over conventional is limited to a small number of patients with breast cancer,^45,46^ it would be worth validating the association between PI composition and immune checkpoint pathway using a second set of tumour samples, ideally acquired from patients treated with immune checkpoint inhibition.

From a technical point of view, this study showed the usefulness of high-resolution MALDI-IMS for describing the alteration of lipid metabolites within tumour tissue. The FA composition of phospholipids in breast cancer has been intensively investigated using classic mass spectrometry approaches.^47,48^ One of the largest studies on breast cancer tissue was conducted by Hilvo et al., who reported that the increase in PCs with a specific FA composition could be a potential biomarker predicting the loss of hormone receptor expression, higher histological grade and poorer prognosis.^49^ Several studies using MALDI-IMS with lower spatial resolution showed similar results.^16,50–52^ In this study, high-resolution MALDI-IMS could distinguish the cancer cell clusters and the surrounding stromal components, which enabled us to identify the accumulation of PI(18:0/20:3) in “cancer cells” as a potential biomarker. A comparison between MALDI-IMS and LC–MS showed that MALDI-IMS exhibits excellent performance in the proportional analysis of PIs. In addition, it showed that the LC–MS analysis was unable to detect the accumulation of PI(18:0/20:3) in cancer cells, further confirming the advantage of MALDI-IMS over conventional mass spectrometry (Supplementary Fig. S1). Considering that laser capture microdissection or cell sorting are potentially incompatible with lipid analysis, MALDI-IMS is one of the best options for identifying cancer cell-related lipid profiles in clinical samples. Since membrane lipid composition determines membrane integrity and influences the sensitivity of cancer cells to cytotoxic drugs such as cisplatin and doxorubicine,^53–56^ multiple drugs targeting lipid metabolism are under development.^57,58^ In particular, metformin can modulate lipid metabolism.^59^ One of the largest drawbacks in developing lipid-modifying drugs for cancer treatment is the absence of an assay to assess their effect on lipid metabolites in each cellular component within the tumours.^60^ In this context, the assessment of lipid distribution using high-resolution MALDI-IMS is a promising way to guide and monitor the intratumoural effects of lipid-modifying drugs. We found that MALDI-IMS is a concise and robust method for analysing phospholipids in clinically acquired tumour samples. Therefore, it would be applicable to high-throughput screening of compounds that could enhance the immune checkpoint inhibition.

Finally, there are limitations of high-resolution MALDI-IMS: firstly, the lipid profiling of single cells is impossible with the current MALDI-IMS resolution. Therefore, higher resolution is necessary to discriminate individual immune cell subsets. Secondly, MALDI-IMS could not cover all lipid species. It is essential to measure the distribution for understanding intratumoural FA mobilisation more precisely. However, it is particularly challenging to assess the distribution of free FAs because of their low molecular weight, low abundance and poor ionisation efficacy.^61^ Thirdly, the quality of imaging in MALDI-IMS can be strongly affected by sample processing. Measurement failure caused by poor processing can happen frequently even when performed by a skilled person. Therefore, it would be ideal to validate the clinical significance of histological PI(18:0/20:3) accumulation by using proper patients’ cohort. However, using our standardised tissue-processing protocol, we achieved a successful measurement rate of more than 96% (56 out of 58 samples, Fig. 1). In addition, the difference in storage method (−80 °C vs. liquid nitrogen) did not affect the result (Table 1). Recently, a concise method for real-time lipid detection in tumour samples, called the probe electrospray ionisation (PESI)/MS system has been developed.^62^ The parallel development of rapid and concise lipid-measuring technology is essential to validate the lipid biomarkers in larger-scale clinical studies.

## Conclusions

High-resolution MALDI-IMS revealed dynamic FA alteration of PIs occurring in cancer cells during tumour progression. Combined gene expression analysis suggested that the sequential conversion of PIs-MUFA to PIs-PUFA affected multiple immune pathways, including the PD-1-related immune checkpoint pathway in primary breast tumours. This study uncovered an unappreciated link between the FA composition of PIs and antitumour immunity. Further investigation on the mechanistic aspects of the PUFA-trafficking system is necessary to better understand the complexity of the regulation of PI composition in breast cancer.

## Supplementary information

Fig S1

Fig S2

Fig S3

Fig S4

Fig S5

Table S1

Table S2

Table S3

Table S4

Table S5

Table S6

## Data Availability

All data generated or analysed during this study are included in the supplementary tables. Alternatively, they are also available from the corresponding authors upon reasonable request.
