# Supplementary material for: High-resolution imaging mass spectrometry combined with transcriptomic analysis identified a link between fatty acid composition of phosphatidylinositols and the immune checkpoint pathway at the primary tumour site of breast cancer
Source: Br J Cancer. 2019 Dec 10;122(2):245–57. doi: 10.1038/s41416-019-0662-8 (PMC7051979; doi:10.1038/s41416-019-0662-8)
Supplement: Supplementary file 1 — Fig S1 [file 41416_2019_662_MOESM1_ESM.pdf]

Fig. S1

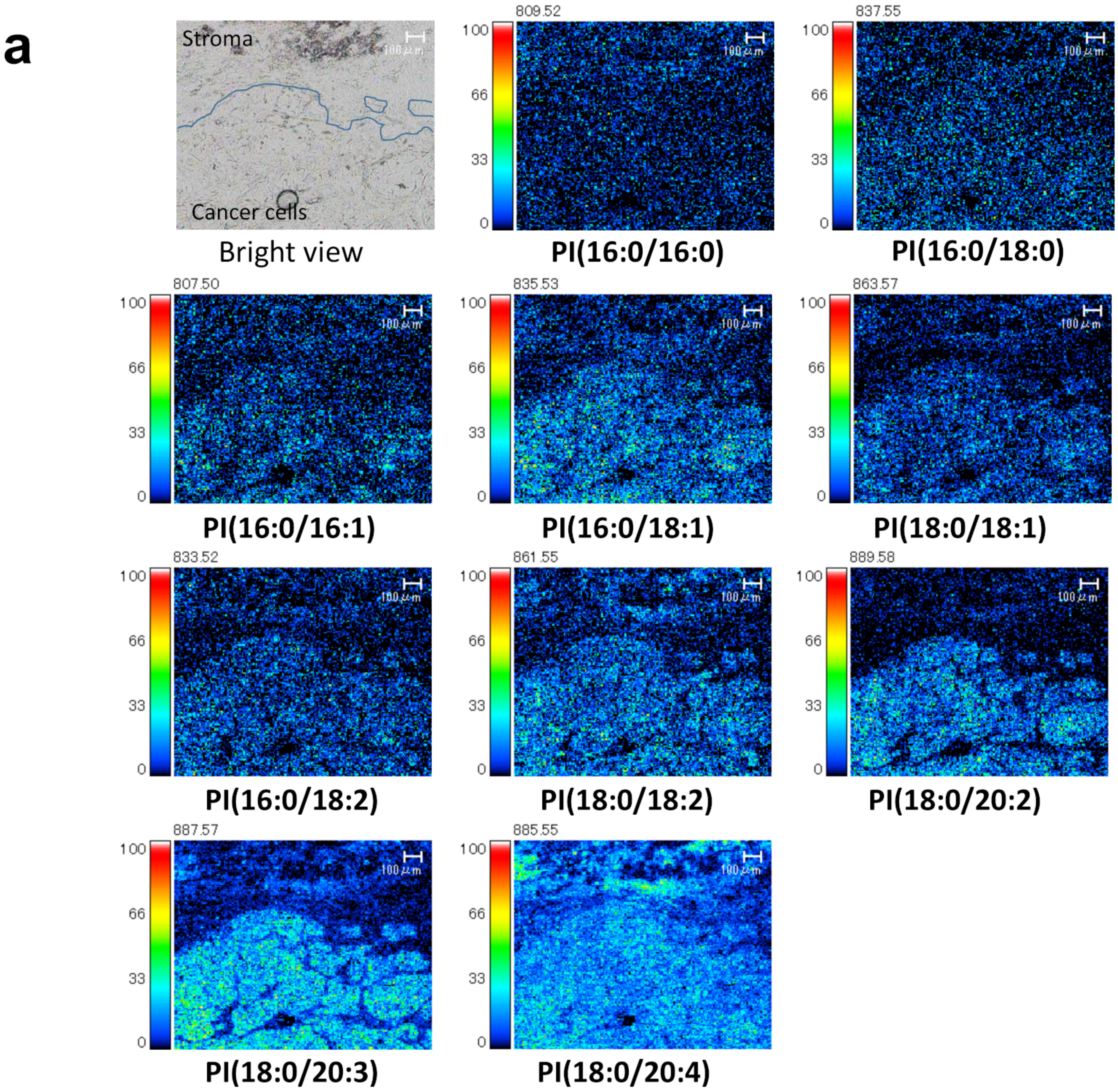

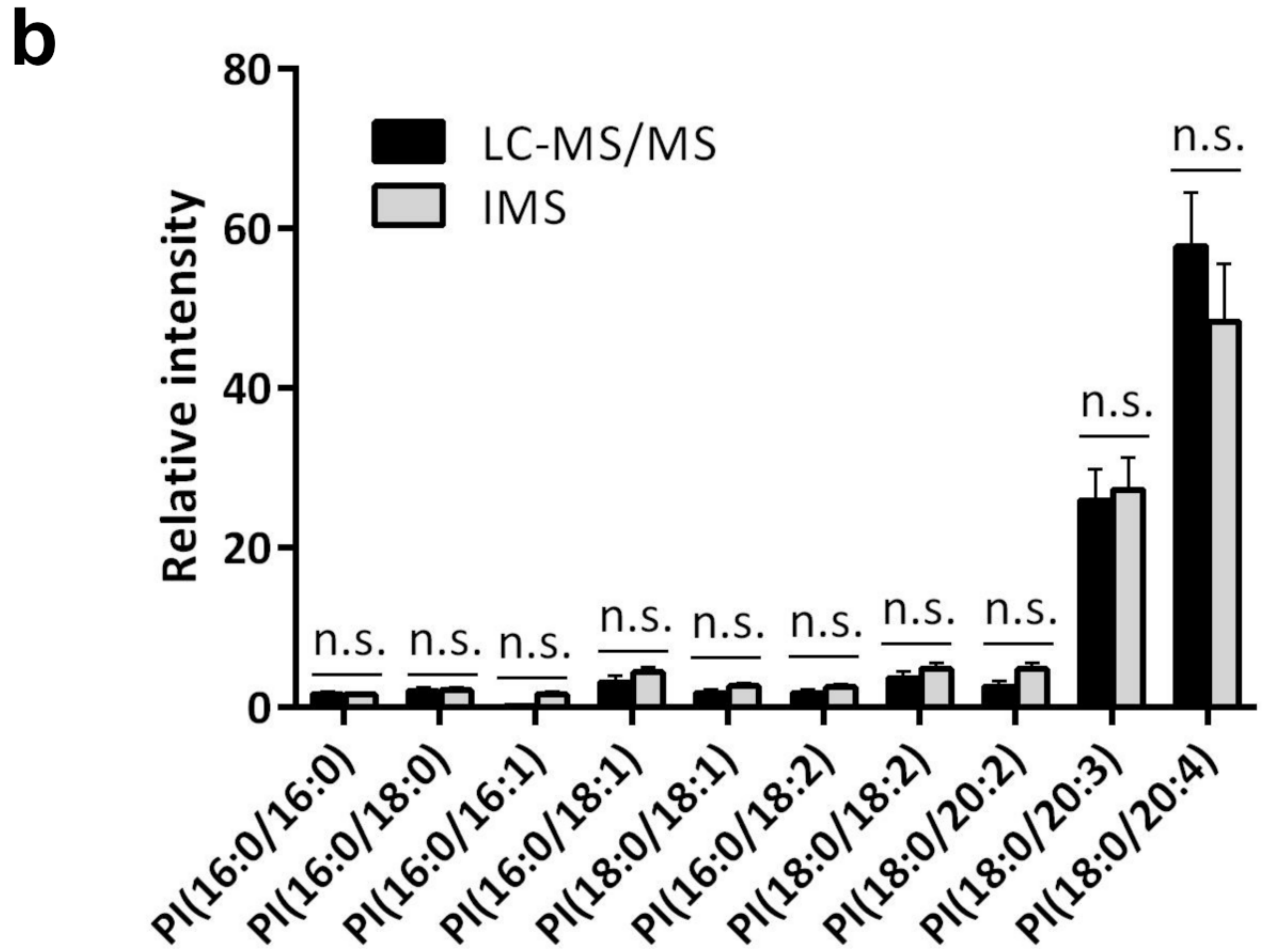

a. histological mapping of PIs in the breast tumor obtained from the patient derived xenograft. Blue line in bright view indicates the border of cancer cell cluster and surrounding stroma. Relative intensity of ion signals is represented by an RGB scale. Scale bar (white): 100 µm. **b.** Comparison between MALDI-IMS and Liquid chromatography (LC)-MS analysis of the same PDX-derived tumor. Two-way ANOVA with Sidak's post hoc multiple comparisons test. Error bar: SD.
