# Supplementary material for: High-resolution imaging mass spectrometry combined with transcriptomic analysis identified a link between fatty acid composition of phosphatidylinositols and the immune checkpoint pathway at the primary tumour site of breast cancer
Source: Br J Cancer. 2019 Dec 10;122(2):245–57. doi: 10.1038/s41416-019-0662-8 (PMC7051979; doi:10.1038/s41416-019-0662-8)
Supplement: Supplementary file 2 — Fig S2 [file 41416_2019_662_MOESM2_ESM.pdf]

Fig. S2

a

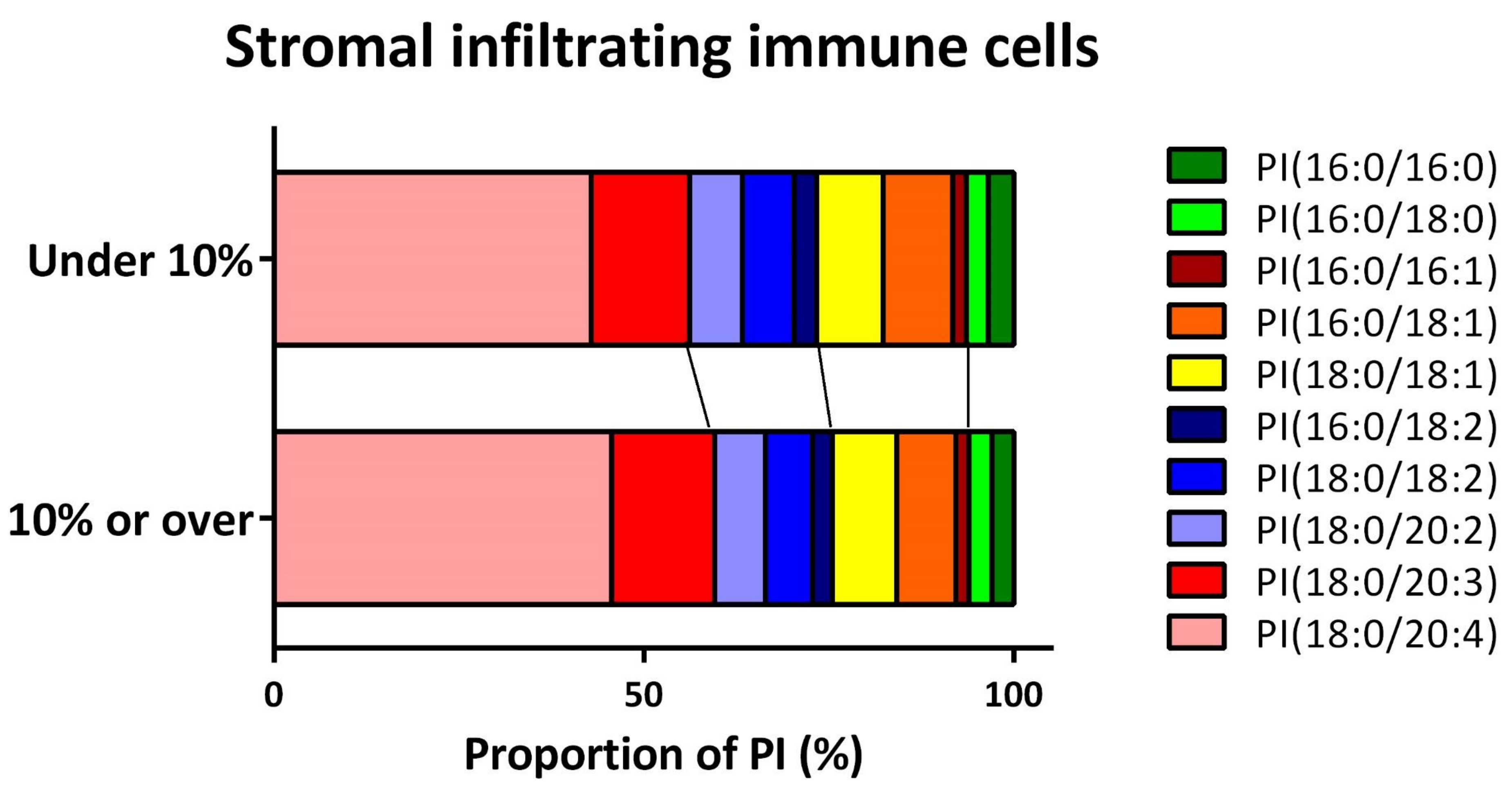

b

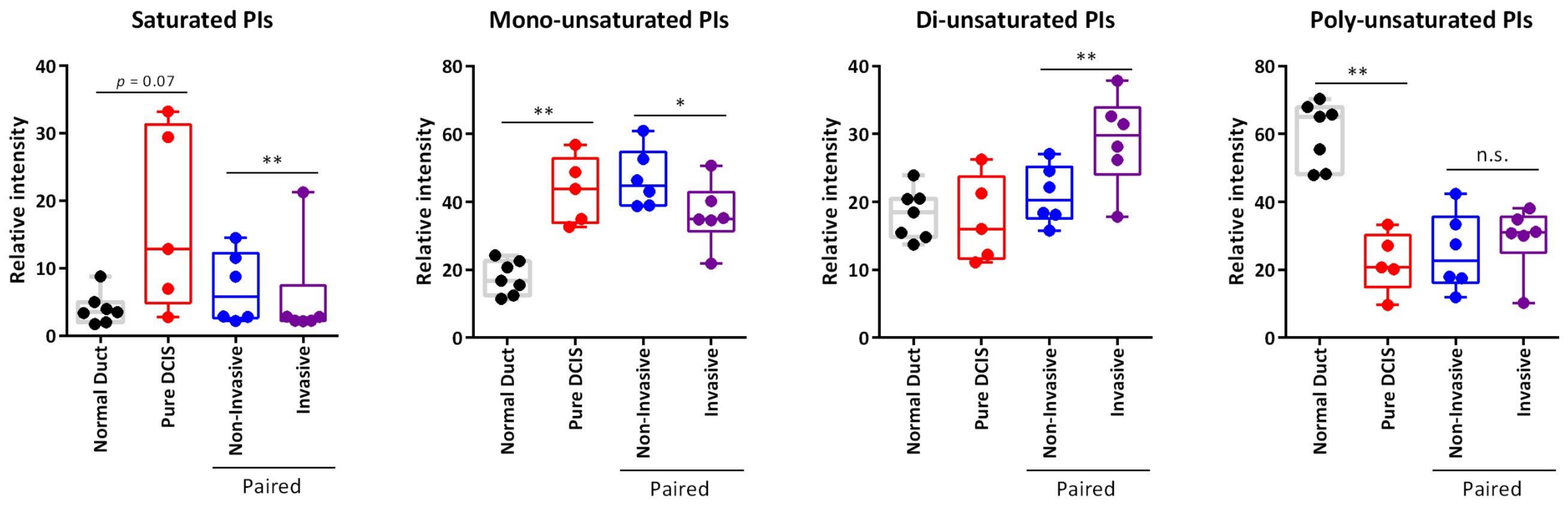

a. the difference of averaged FA composition of PIs between the stroma consisting of < 10% immune cells and the stroma consisting of ≥10% immune cell infiltration. There is no statistically significant difference between 2 groups. Two-way ANOVA with Sidak's post hoc multiple comparisons test was used. **b**. comparison of relative intensities of PIs at the different stage of cancer progression. Pure DCIS (red) means non-invasive cancer cells accompanying no invasive diseases. Non-invasive cancer cells (blue) and invasive cancer cells (purple) are appeared in the same tumor section. * indicates a statistically significant difference *p < 0.05; **p < 0.01; n.s.: not significant.
