# Supplementary material for: High-resolution imaging mass spectrometry combined with transcriptomic analysis identified a link between fatty acid composition of phosphatidylinositols and the immune checkpoint pathway at the primary tumour site of breast cancer
Source: Br J Cancer. 2019 Dec 10;122(2):245–57. doi: 10.1038/s41416-019-0662-8 (PMC7051979; doi:10.1038/s41416-019-0662-8)
Supplement: Supplementary file 3 — Fig S3 [file 41416_2019_662_MOESM3_ESM.pdf]

Fig. S3

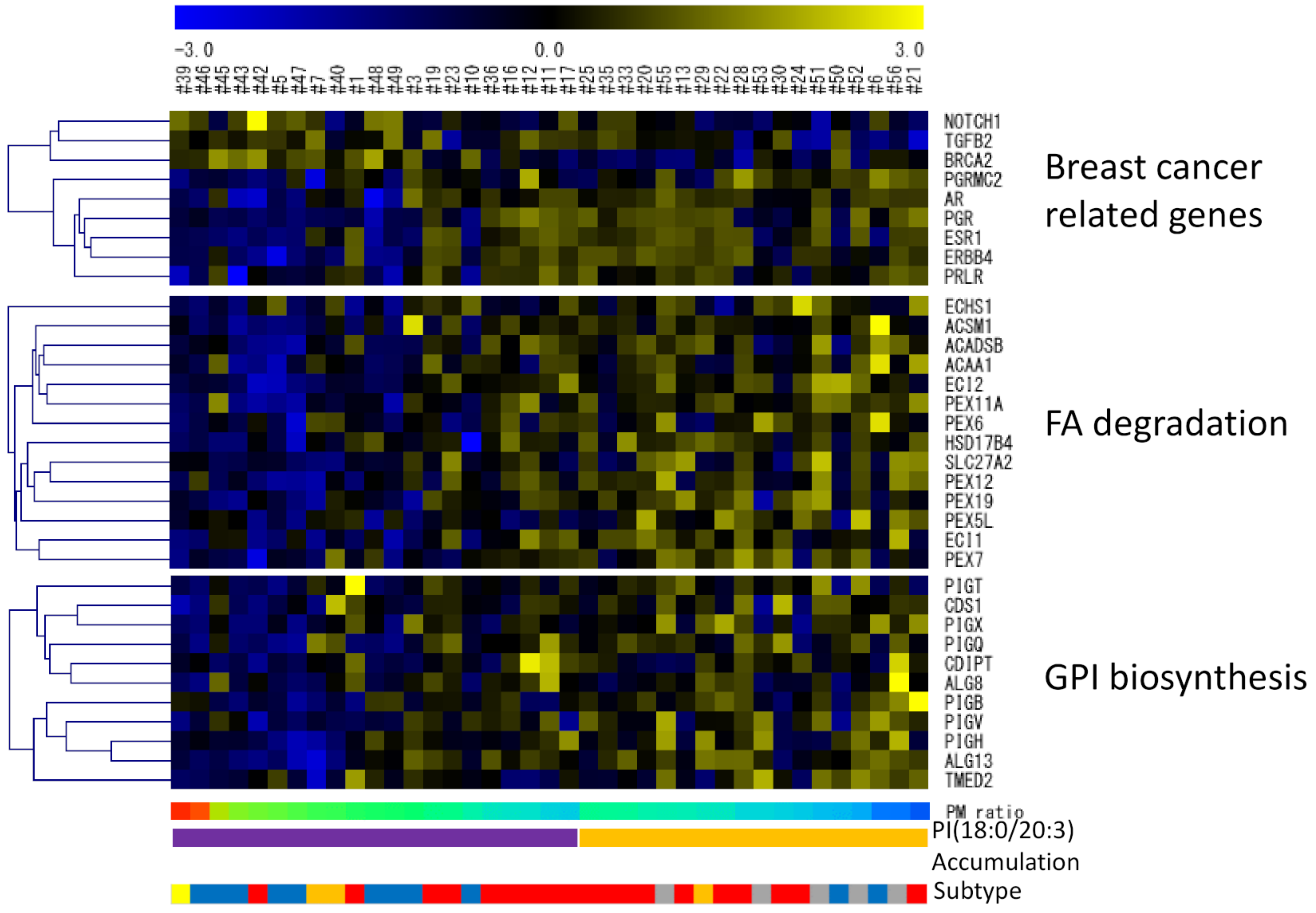

Heat maps illustrating the expression of genes related to breast cancer, fatty acid (FA) degradation and glycosylphosphatidylinositol (GPI) biosynthesis. PM ratio: the ratio of PIs-PUFAs (PI[18:0/20:3] and PI[18:0/20:4]) to PIs-MUFAs (PI[16:0/18:1] and PI[18:0/18:1]). Gene expression values are indicated by blue to yellow color scale (top bar). Presence and absence of PI(18:0/20:3) accumulation in histological images are shown in purple and yellow, respectively. The values of PM ratio are indicated by rainbow color scale. Tumor subtypes are shown in red (luminal), blue (triple negative), orange (luminal-Her2), yellow (Her2), and gray (pure DCIS). (bottom bar).
