# Supplementary material for: High-resolution imaging mass spectrometry combined with transcriptomic analysis identified a link between fatty acid composition of phosphatidylinositols and the immune checkpoint pathway at the primary tumour site of breast cancer
Source: Br J Cancer. 2019 Dec 10;122(2):245–57. doi: 10.1038/s41416-019-0662-8 (PMC7051979; doi:10.1038/s41416-019-0662-8)
Supplement: Supplementary file 4 — Fig S4 [file 41416_2019_662_MOESM4_ESM.pdf]

Fig. S4

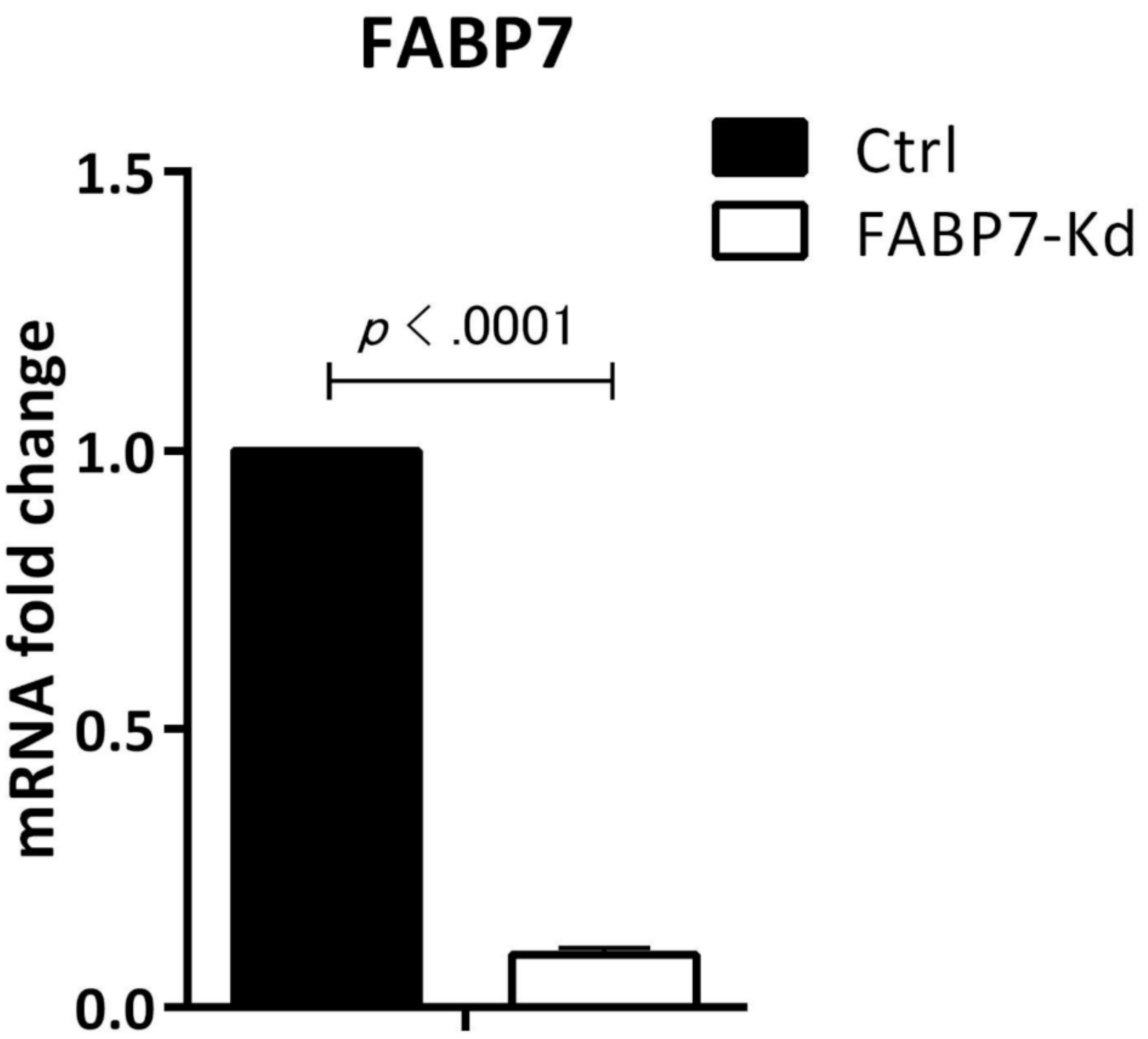

The expression of FABP7 in control cells and FABP7-knock down cells are shown. Student *t*-test was carried out test the difference. Real-time PCR was performed on a 7900HT Fast Real Time PCR System (Applied Biosystems) using the comparative threshold cycle method. Expression data were normalized to the expression of the two control genes: ACTB and HPRT1. Primer sequences were follows; ACTB forward: ATTGGCAATGAGCGGTTC; ACTB reverse: GGATGCCACAGGACTCCAT; HPRT1 forward: CCAGTCAACAGGGGACATAAA; HPRT1 reverse: CACAATCAAGACATTCTTTCCAGT; FABP7 forward: TGAAACCACTGCAGATGATAGAA; FABP7 reverse: TTTCTTTGCCATCCCATTTC.
