# Supplementary material for: High-resolution imaging mass spectrometry combined with transcriptomic analysis identified a link between fatty acid composition of phosphatidylinositols and the immune checkpoint pathway at the primary tumour site of breast cancer
Source: Br J Cancer. 2019 Dec 10;122(2):245–57. doi: 10.1038/s41416-019-0662-8 (PMC7051979; doi:10.1038/s41416-019-0662-8)
Supplement: Supplementary file 5 — Fig S5 [file 41416_2019_662_MOESM5_ESM.pdf]

Fig. S5

a

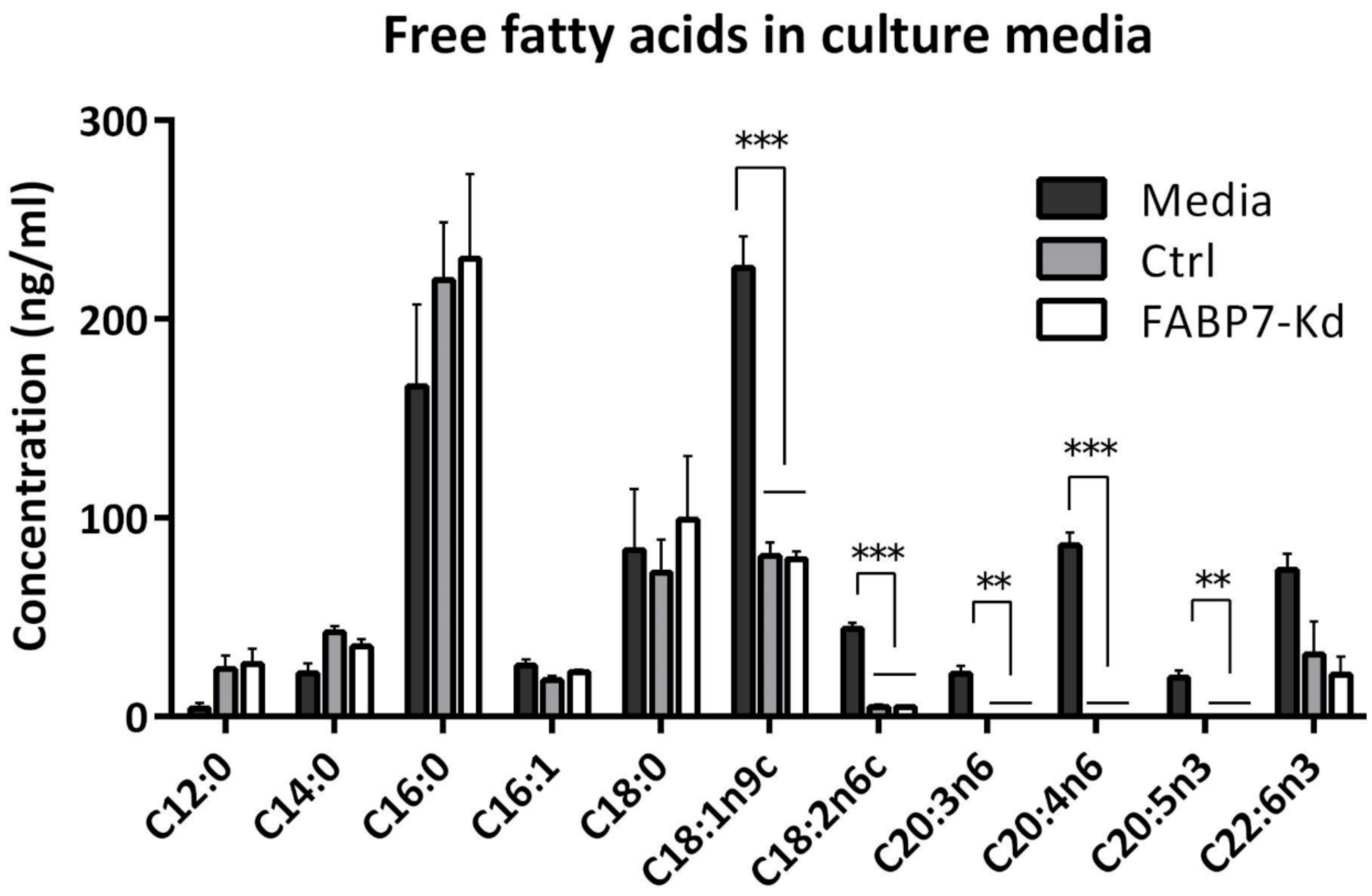

b

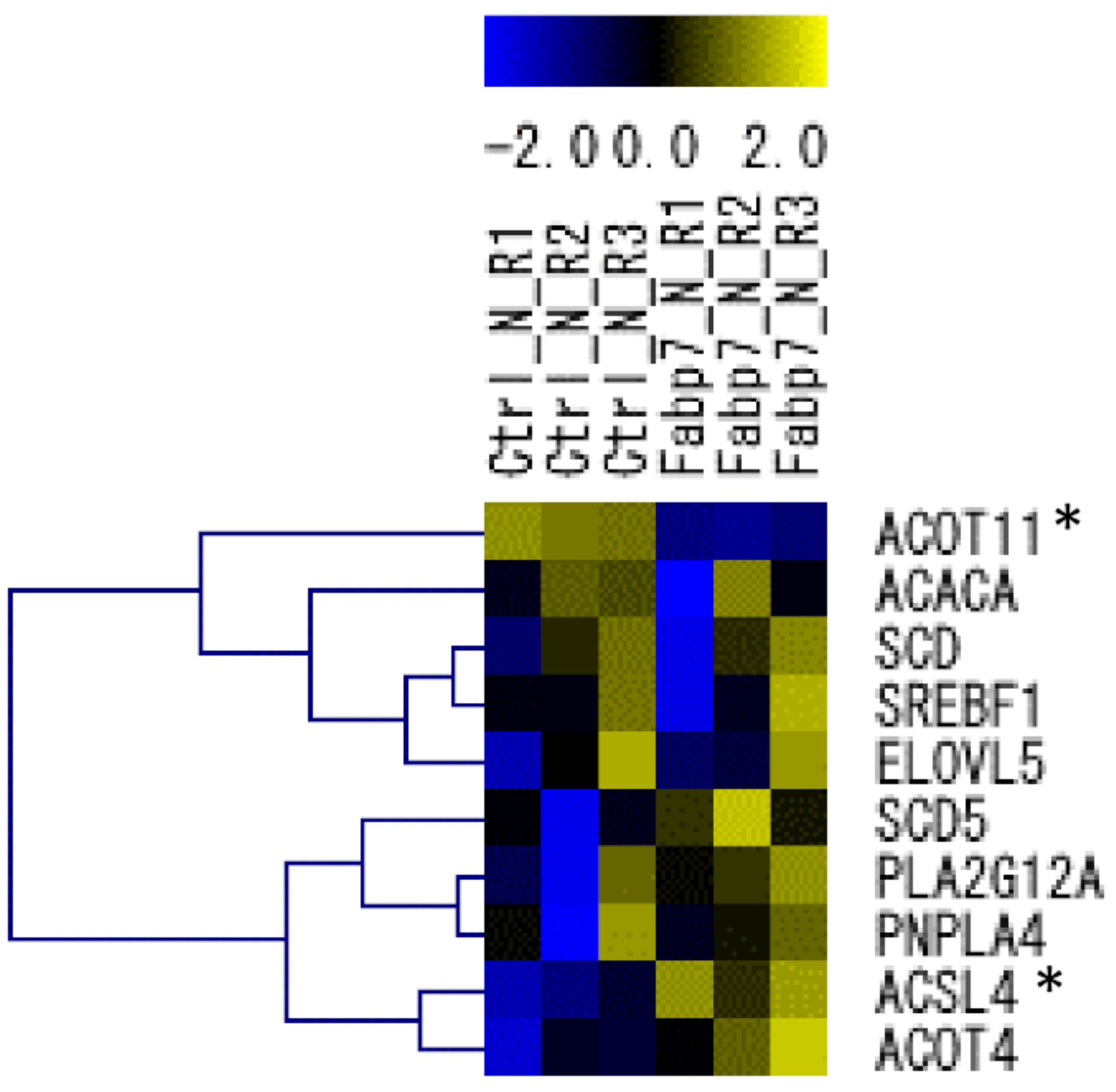

a. the difference of FA composition in culture media. The media without cells (black), the media of control cells (gray) and the media of FABP7 knockdown cells (white) were shown. * indicates a statistically significant difference in the comparison Two-way ANOVA with Sidak's post hoc multiple comparisons test. **$p < 0.01$; ***$p < 0.001$. **b**. a heat map illustrating the expression of individual genes related to “Unsaturated fatty acid synthesis and transport”. Gene expression values are indicated by a blue to yellow color scale (top bar). The analysis was performed in biological triplicate. * indicates a statistically significant difference.
